# The loss of transcriptional inhibition by the photoreceptor-cell specific nuclear receptor (NR2E3) is not a necessary cause of enhanced S-cone syndrome

**Published:** 2007-04-06

**Authors:** Mathias Fradot, Olivier Lorentz, Jean-Marie Wurtz, José-Alain Sahel, Thierry Léveillard

**Affiliations:** 1 Inserm U592 Université Pierre et Marie Curie, Laboratoire de Physiopathologie Cellulaire et Moléculaire de la Rétine, Hôpital Saint-Antoine, Paris, France; 2 Departement de Biologie et de Génomique Structurales, IGBMC, CNRS/Inserm/Université Louis Pasteur, BP10142, Illkirch Cedex, France; 3 University College of London, Institute of Ophthalmology, UK

## Abstract

**Purpose:**

To investigate functional consequence on photoreceptor-cell specific nuclear receptor (NR2E3) transcriptional activity of enhanced S-cone syndrome (ESCS) mutations localized in ligand binding domain (LBD).

**Methods:**

Point mutations were introduced into the LBD of full length and Gal4 chimeric NR2E3 receptors and transcriptional activity was investigated by using transient co-transfection assay on corresponding luciferase reporters. Expression and DNA binding properties of transfected mutant and wild-type receptors were tested by Western blotting and gel shift assay.

**Results:**

Our analysis show that two ESCS mutations, missense mutations R385P and M407K, abolished NR2E3 repressive activity in the context of full-length and Gal4 chimeric receptors, while W234S and R311Q mutants retained their repressive activity in both assays. All mutant receptors maintained their stability and DNA binding ability.

**Conclusions:**

These results showed that NR2E3 mutations localized in LBD induce ESCS disease without affecting inhibitory activity as recorded in vitro. This demonstrates the absence of correlation between transcriptional inhibition and ESCS phenotype. This analysis suggests that NR2E3 might have transcriptional activation properties not yet identified.

## Introduction

Enhanced S-cone syndrome (ESCS) is an autosomal recessive retinopathy characterized by a gain of visual function: Patients with ESCS exhibit electroretinogram (ERG) responses to short wavelength stimulation 30 times higher in amplitude than normal corresponding to S-cone function. This disease also includes night blindness and similar photopic and scotopic ERGs [1,2]. Histological data from one elderly patient with advanced disease showed an absence of rods but a two-fold increase in the cone population, 92% of which were thought to be S-cones, while in vivo examination of early disease has shown an abnormal retinal architecture [3,4]. These histological findings would explain the gain of function observed in ESCS patients. ESCS could therefore be considered as a developmental defect, which results in an increase in photoreceptor cells taking an S-cone fate [5].

Identification of mutations in the *NR2E3* gene, encoding an orphan nuclear receptor transcription factor, has paved the way to our current understanding of the disease [6,7]. The expression of NR2E3 is restricted to rod photoreceptors in human, although the disease is characterized by an increase in S-cone function [7-9].

Studies of *rd7* mouse retinas, a murine model with a homozygous insertion of a L1 retrotransposon into exon 5 of *Nr2e3* gene, have also revealed a two fold increase in S-cone number, retinal dystrophy at early stages and slow retinal degeneration [10-12]. Expression of NR2E3 in mouse retina is restricted to rod nuclei, starts after the completion of cone cell birth, and peaks after completion of rod cell differentiation [13,14].

The current hypothesis is that NR2E3 represses S-cone fate as well as participates in rod photoreceptor commitment [5,13-17]. The intrinsic genetic program appears to be the major determinant of cell-fate commitment in the retina [18]. The competence model of cell-fate determination proposes that a homogeneous pool of multipotent progenitors passes through states of competence where it can produce a given set of cell types [19]. Transcription factors are among the best characterized intrinsic factors, and NR2E3 may have a similar role as its paralog NR2E1 in driving pluripotent cells to a particular fate [20].

NR2E3, as a nuclear receptor, possesses a central DNA binding domain (DBD), and a C-terminal ligand binding domain (LBD) [21]; it was originally described as a transcriptional repressor and binds DNA as a homodimer [14,22]. Physical and functional interactions of NR2E3 with several transcription factors involved in photoreceptor differentiation have been established [14,23]. It has recently been shown that NR2E3 directly interacts with the nuclear receptor NR1D1 and the homeoprotein Crx [14,23]. These interactions lead to enhanced expression of rod-specific genes and reduced expression of cone-specific genes in vitro. NR2E3 also interacts with Nrl, a photoreceptor specific transcription factor, and modulates its transcriptional activity [15]. The absence of functional *Nrl* gene in mouse gives a severe phenotype with a complete loss of rods replaced by S-cones. Interestingly, the expression of NR2E3 has been shown to be dependent upon Nrl, suggesting that the increase in S-cones in the *Nrl*-/- mouse results in part from the absence of expression of NR2E3 [16].

Analysis of gene expression modification in *rd7* mouse retina has been performed using different approaches [14,15,17]. Microarray analyses revealed an up-regulation of numerous cone-specific genes in *rd7* mouse retina, pointing out NR2E3 repressive function [14,17], while chromatin immunoprecipitation assays associated with reverse transcriptase polymerase chain reaction (RT-PCR) analysis, demonstrated that NR2E3 represses cone specific genes but activates the expression of rod-specific genes [15]. In addition, the transcriptome of the retina of transgenic mice overexpressing NR2E3 confirms the role of NR2E3 as a suppressor of the expression of cone-specific genes [24]. Corbo and Cepko also reported a delay of rhodopsin expression in *rd7* mouse [17]. In rat, during development, there is about a week-long period between birth of rods and onset of rhodopsin expression [25]. During this period, NR2E3 would suppress S-cone fate by reducing S-cone gene expression as well as promoting rod fate by activating rod-specific promoters [14,15,23,24].

In the present paper, we analyzed the transcriptional properties of the LBD of NR2E3 (from residue 113 to 410) mutants found in ESCS, fused to a heterologous DBD (Gal4^DBD^), to circumvent a problem due to DNA binding specificity [26], as well as the full length protein to get a better understanding of the activity of NR2E3 mutants in a more physiological context [14].

We confirmed that NR2E3 inhibitory properties involve the helix H12 of the LBD as observed for other nuclear receptors [14,27]. We reported an absence of correlation between transcriptional inhibitory properties of NR2E3^LBD^ and ESCS, implying the existence of some transcriptional activation properties that might be controlled by a yet to be identified ligand [28].

## Methods

### Transfection assays in COS-1 and HeLa cells

Transfections were performed in COS-1 and HeLa cells, which were maintained in Dulbecco's modified Eagle's medium (DMEM; Gibco, Cergy-Pontoise, France) supplemented with 10% fetal bovine serum (Gibco).

COS-1 cells were transiently transfected by the calcium phosphate precipitate method [29]: Cells were plated at a density of 3.5X 10^5^ cells/ml in 24-well tissue culture plates (500 μl/well) and incubated for 2 h at 37 °C in a humidified 5% CO_2_ incubator before transfection. Cells were transfected with 500 ng of Gal4 or NR2E3 responsive luciferase reporter construct [14], 10 ng of pRL-TK internal reporter construct (Promega, Charbonnieres, France) and a variable amount of different expression constructs. One day after transfection, cells were washed with medium without serum and changed to fresh medium. Two days after transfection, lysates were collected and luciferase activity was measured using the Dual Luciferase Reporter Assay System (Promega).

HeLa cells were transfected using Lipofectamine 2000 reagent (Gibco-BRL). Cells were plated in six-well tissue culture plates (2 μl/well) and left until they reached 80% confluence. Before transfection, cells were washed and changed to OPTI-MEM medium (Gibco-BRL). Cells were transfected with 750 ng of Gal4-responsive luciferase reporter construct, 10 ng of pRL-TK internal reporter construct, a variable amount of different expression constructs and 2.5 μl of Lipofectamine 2000 according to the manufacturer's instructions. Two days after transfection, lysates were collected and luciferase activity was measured using the Dual Luciferase Reporter Assay System. All transfection assays were performed in triplicate. Each assay group was repeated at least twice.

### Site-directed mutagenesis

Point mutations were introduced in NR2E3^LBD^ from pCMV-Gal4-NR2E3^LBD^ and pCMX-HA-NR2E3 constructs obtained from Dr. Mime Kobayashi [6]. Mutations were introduced by oligonucleotide-directed mutagenesis using the thermostable Deep Vent DNA polymerase (New England Biolabs Inc., Beverly, MA). Amplified DNA was digested by *Dpn*I (New England Biolabs Inc.) and used to transform XL-10 Gold ultra-competent *E. coli* cells (Stratagene Europe, Hogehilweg, Netherlands). Mutations were confirmed by sequencing.

### Nuclear protein extraction

Nuclear extracts were prepared from transiently transfected COS-1 cells. Transfected cells were rinsed with 1X PBS, harvested by centrifugation for 5 min at 800x g at 4 °C, and washed with 5 volumes of hypotonic buffer (10 mM HEPES, pH 7.5, 1X complete protease inhibitor cocktail [Boerhinger Mannheim, Mannheim, Germany], 10 mM KCl, 1.5 mM MgCl_2_, 0.5 mM DTT). The cells were suspended with 3 volumes of hypotonic buffer, and incubated for 10 min on ice. Cytoplasmic membranes were disrupted with a pestle B. Nuclei were harvested by centrifugation for 15 min at 1,200x g, suspended with 0.5 volume of low salt buffer (20 mM HEPES, pH 7.5, 1X Complete protease inhibitor cocktail, 2 mM KCl, 1.5 mM MgCl_2_, 0.2 mM EDTA, 0.5 mM DTT, 25% glycerol) before the disruption of nuclear membranes by drop-wise addition of 0.5 volume of high salt buffer (20 mM HEPES, pH 7.5, 1X Complete protease inhibitor cocktail, 1.2 M KCl, 1.5 mM MgCl_2_, 0.2 mM EDTA, 0.5 mM DTT, 25% glycerol). Nuclear lysates were incubated 30 min on ice under agitation and harvested by centrifugation for 30 min at 16,000x g at 4 °C. Supernatants were aliquoted and stored at -80 °C until assayed.

### NR2E3 protein binding assays

NR2E3 protein binding was detected by electrophoretic mobility shift assays. DNA probes containing Gal4 (5'-GGG CCG ACG GGT GAC AGC CCT CCG ACG GCC C-3') and Kni x2 binding site (5'-TAA CCT TTT AAA AGT CAA AAG TCA ACT TCC AAC-3') [6] were prepared by annealing complementary oligonucleotides and labeled at both end with [α-^32^P]dCTP (6,000 Ci/mmol; Amersham Biosciences, Saclay, France) by filling with the large fragment of DNA polymerase I (Promega). Binding reactions contained 40,000 cpm of probe, 1 μg of double stranded poly(dI-dC) (Amersham Biosciences), and 12.5 μg of bovine serum albumin in 50 μl of binding buffer (25 mM HEPES, pH 7.5, 50 mM KCl, 5 mM MgCl_2_, 0.1 mM EDTA, 0.1% NP40, 10% glycerol), with the nuclear protein fraction and competitor oligonucleotide, 100 fold excess, as indicated. Reaction mixtures were incubated 40 min at room temperature, and protein-DNA complexes were separated by electrophoresis on 4% polyacrylamide gels in 0.5X Tris-borate-EDTA buffer at room temperature. Radioactivity in gels was detected by autoradiography.

### Western blot

Nuclear extracts from transiently transfected COS-1 cells were analyzed by Western-blot. Protein concentrations were estimated by Bradford's technique. Proteins (10 μg/lane) were separated by 10% SDS-PAGE and transferred onto nitrocellulose membrane (Schleicher and Schuell, Mantes-la-Ville, France), blocked with non-fat milk (5% w/v) and incubated with the appropriated primary antibody overnight at 4 °C. Gal4-NR2E3^LBD^ fusion proteins were detected using anti-Gal4^DBD^ mouse monoclonal antibody (1/2,000, gift from Pierre Chambon, Ph.D. France) while NR2E3 full length proteins were detected using anti-HA-Tag mouse monoclonal antibody (1/1,000, Cell Signaling Technology Inc., Danvers, MA). The membrane was then washed, incubated with horseradish peroxidase labeled secondary antibody (1/15,000, Jackson ImmunoResearch Laboratories Inc., West Grove, PA), and immunoreactive bands were detected by enhanced chemiluminescence (ECL+; Amersham, Biosciences).

## Results

### Analysis of transcriptional repression by NR2E3^LBD^

**Figure 1:**
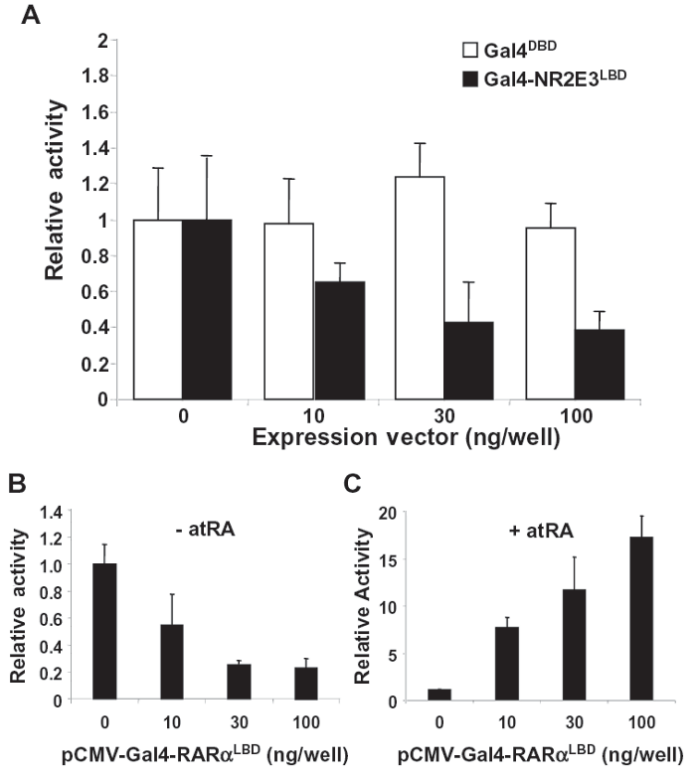
Transcriptional repression by NR2E3^LBD^. COS-1 cells were transiently transfected with Gal4^DBD^ or Gal4-NR2E3^LBD^ expression plasmids. Transcriptional activity was measured for the different luciferase Gal4-reporter genes. **A**: Luciferase activity in the presence of an increasing amont of GAL-NR2E3^LBD^. **B**: Luciferase acitity in the presence of an increasing amount of GAL-RARα^LBD^. **C**: Luciferase activity in the presence of an increasing amount of GAL-RARα^LBD^ in the presence of its ligand RA. Normalized values are expressed as relative luciferase activity.

In order to study the transcriptional properties of the LBD of NR2E3, we expressed it as a chimeric protein, fused to the DBD of the yeast transcription activator Gal4. This construct was transiently transfected into COS-1 cells and tested on Gal4-responsive reporter plasmids using luciferase assay (Figure 1A). First, to verify that the inhibitory properties of Gal4-NR2E3^LBD^ reported by others [14,22] were not due to specific elements in the promoter, we performed experiments using Gal4 binding sites, upstream of two different minimal promoters; the β-globin and the SV40 proximal promoter. In Figure 1A, transcriptional inhibition mediated by Gal4-NR2E3^LBD^ was dose-dependent from 10-100 ng for both reporter constructs. This inhibition is due to the NR2E3^LBD^, since the heterologous DBD alone did not significantly inhibit the expression of luciferase at 10 and 30 ng and was inhibitory only for the highest amount of expression vector used (100 ng) with the SV40 promoter. The activity was similar to that obtained with another nuclear receptor, RARα, tested under the same conditions, and in absence of its ligand (Figure 1B). This inhibition was reverted in presence of RAR ligand, the all-trans retinoic acid (Figure 1C).

### Mutational analysis of NR2E3^LBD^

**Figure 2:**
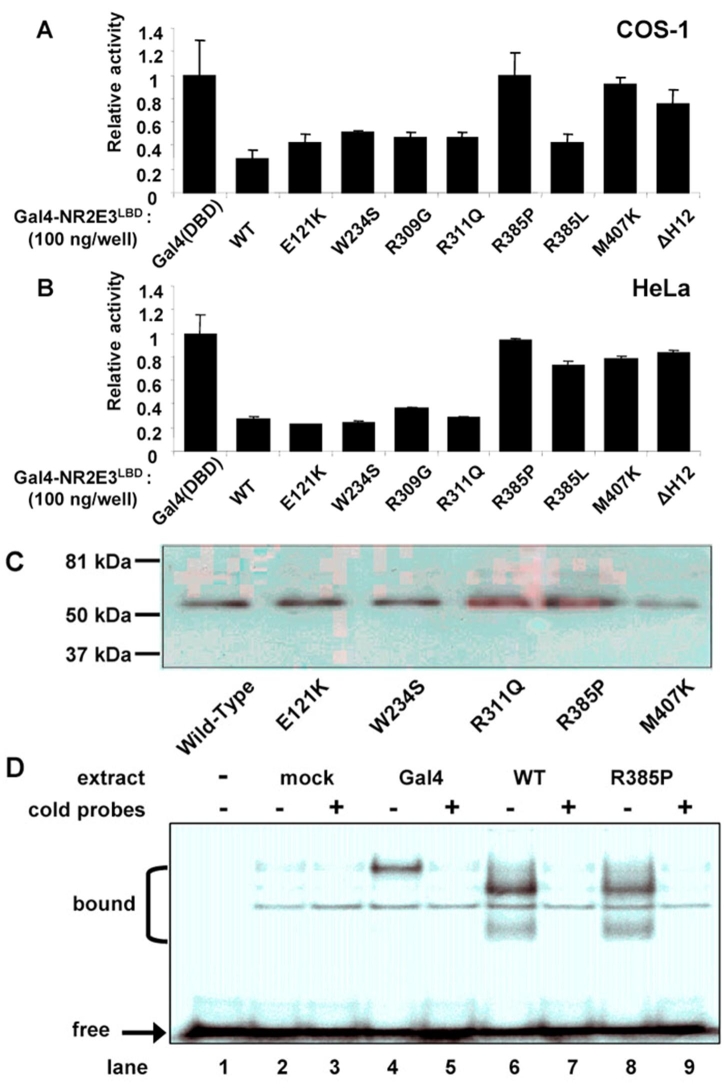
Transcriptional effect of NR2E3 mutations on Gal4 chimeric receptor. **A**: COS-1 cells were transiently transfected with 100 ng of Gal4-NR2E3^LBD^ wild-type and mutant expression plasmids. Transcriptional activity of a Gal4 responsive reporter gene was measured. **B**: HeLa cells were transiently transfected with 100 ng of Gal4-NR2E3^LBD^ wild-type and mutant expression plasmids, and transcriptional activity of a Gal4 responsive reporter gene was measured. Normalized values are expressed as relative luciferase activity. **C**: Expression of Gal4-NR2E3^LBD^ wild-type and mutants in COS-1 transfected cells. **D**: Electrophoretic mobility shift assay of Gal4 full-length, wild-type (WT), and R385P mutated Gal4-NR2E3^LBD^ using a Gal4 probe. Bound indicates the different shifted bands, and free denotes unbound probe.

To examine the functional consequences of NR2E3 mutations described in ESCS, we introduced six mutations found in ESCS (E121K, W234S, R309G, R311Q, R385P, and M407K) and two artificially designed mutations (R385L and αH12) into the Gal4-chimeric receptor by site-directed mutagenesis. Most mutations examined are localized in the LBD, between positions 163 and 410, of the human NR2E3 protein [7]. These Gal4-NR2E3^LBD^ mutant constructs were transfected into COS-1 cells and tested for their ability to repress transcription from a Gal4 responsive element fused to the β-globin minimal promoter (Figure 2A). Four out of six of the ESCS mutations (E121K, W234S, R309G and R311Q) had slightly reduced inhibitory activity as compared to wild-type. For these mutants, there was no correlation between the NR2E3^LBD^ transcriptional inhibition activity and the ESCS phenotype.

As described previously, and confirmed here in another cell line, the deletion of mutant H12 (N397Stop) results in the total absence of transcriptional repression [14]. This is in agreement with others studies where deletion of helix H12 enhances repression and co-repressor binding, although several nuclear receptors lacking helix H12 act as transcriptional repressor [30-32].

The ESCS mutation M407K corresponds to a position in the helix H12 of nuclear receptors known to modulate the affinity of the LBD to co-regulators. Again, and as seen by others, the M407K NR2E3 mutant protein is not able to mediate transcriptional repression [14].

The ESCS R385P mutation also results in loss of inhibition. This mutant is not localized within the helix H12, and the loss of transcriptional inhibitory property must result from a distinct mechanism. In order to test the possibility of a conformational constraint that might be created by the proline residue, we designed the artificial R385L mutant with an arginine residue replaced by a leucine residue. The R385L mutant has an inhibitory activity slightly weaker than of the wild-type NR2E3 and similar to that of four ESCS mutants.

Mutational analysis was also performed in HeLa cells using Gal4 chimeric receptors and Gal4 responsive reporters (Figure 2B). The six examined ESCS mutations presented similar behavior in HeLa and COS-1 cells; four of them (E121K, W234S, R309G, and R311Q) retained a slightly reduced inhibitory activity, while R385P and M407K mutants were not able to mediate transcriptional inhibition in HeLa cells. Only the R385L artificial mutant has different behavior in the two cell lines. It lost its repressive activity in COS-1 but remained active in HeLa cells. The αH12 mutant displayed similar activity in both cell lines.

In order to check that the lack of transcriptional inhibition was not the result of a difference in protein stability in COS-1 cells, we performed Western blotting analysis (Figure 2C). The mutant fusion proteins were confirmed to be expressed at similar levels. The R385P mutant, which lacks the inhibitory activity, was even expressed at a slightly higher level than the wild-type construct. The loss of activity of the R385P mutant could also theoretically result from misfolding of the protein, and the resulting inability of this mutant to bind the Gal4 responsive element. To study this hypothesis, nuclear extracts from COS-1 transfected cells were prepared and used in gel mobility assays in conjunction with oligonucleotides corresponding to the Gal4 binding site (Figure 2D). Gal4 protein, used as a positive control, gave a shift in agreement with its molecular weight (lane 4 and 5). Wild-type Gal4-NR2E3^LBD^ displayed two bands shifted in mobility (lanes 6 and 7) that were also observed when the R385P mutant protein extract was used (lanes 8 and 9). This provides the evidence that R385P mutation has no effect on the conformation of the heterologous DNA binding domain.

### Mutational analysis of full-length NR2E3

**Figure 3:**
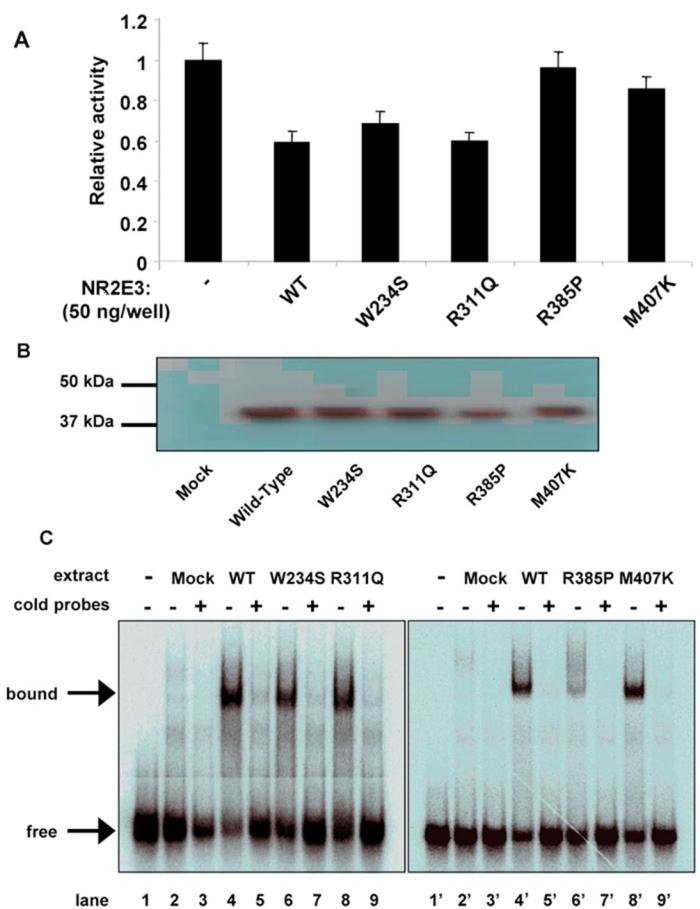
Transcriptional effect of NR2E3 mutations on full-length receptor. **A**: COS-1 cells were transiently transfected with 100 ng of NR2E3 wild-type and mutant expression plasmids, and transcriptional activity of a NR2E3 responsive reporter gene was measured. Normalized values are expressed as relative luciferase activity. **B**: Expression of NR2E3 wild-type and mutants in COS-1 transfected cells. **C**: Electrophoretic mobility shift assay of full-length wild-type (WT) and mutated NR2E3 using a Kni x2 probe [6]. Bound indicates shifted bands, and free denotes unbound probe.

Functional consequences of NR2E3 mutations were also examined in full-length protein. Four mutations found in ESCS (W234S, R311Q, R385P, and M407K) were introduced into HA-tagged full-length NR2E3 by site directed mutagenesis. These mutated NR2E3 constructs were transfected into COS-1 cells and tested for their ability to repress transcription from a NR2E3 responsive element fused to the thymidine kinase minimal promoter (Figure 3A) [7,14]. Two out of four of the mutations (W234S and R311Q) had slightly reduced inhibitory activity as compared to wild-type, while R385P and M407K mutants lost most of their repressive activity.

**Figure 4:**
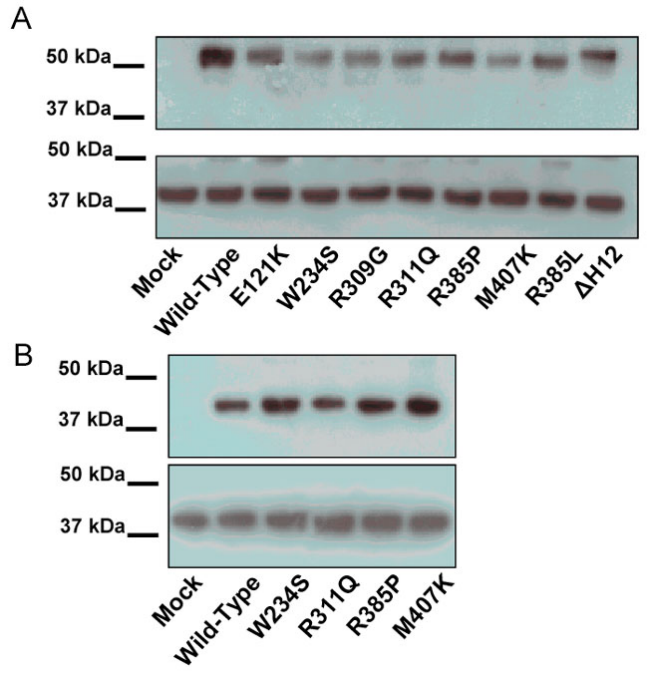
Expression of Gal4-NR2E3^LBD^. **A** and **B** mutant proteins after transfection into COS-1 cells. The bottom panels represents β-actin loading controls. **A**: Western blotting against the GAL4-NR2E3 fusion proteins. **B**:L Western blotting against the hemaglutinin antigene (HA) tagged NR2E3 proteins.

To check for the stability of NR2E3 mutant proteins, we analyzed COS-1 transfected cells by Western blotting. All the full-length receptors, mutant or wild-type, were expressed at similar level and had the expected electrophoretic mobility (Figure 3B and Figure 4). As no natural DNA response element has been identified for NR2E3, we used Kni 2X2, a dimeric response element, which NR2E3 is able to bind [6,14]. Dimerization of several nuclear receptors has been shown to be dependant upon LBD, indicating that LBD-localized mutations could affect DNA binding ability. Nuclear extracts from COS-1 transfected were analyzed by gel-shift mobility with oligonucleotides corresponding to Kni X2 response element [6] in order to check for the DNA binding of the different NR2E3 proteins; mutant and wild-type (Figure 3C). All the mutated full-length proteins displayed a band (lanes 6, 6', 8, and 8') that was also observed with the wild-type full-length protein (lanes 4 and 4'). This provides evidence that NR2E3 dimerization ability was not affected by these ESCS mutations.

**Figure 5:**
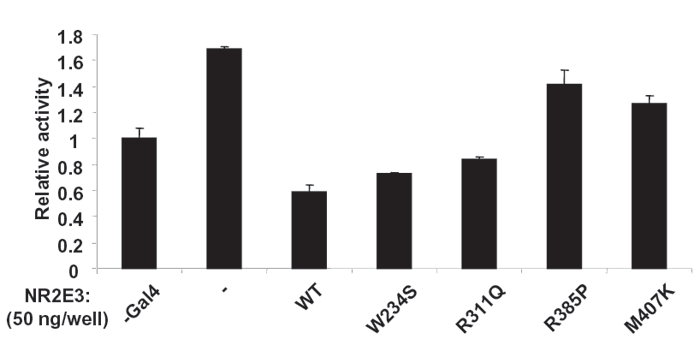
Repression of Gal4 activated promoter by NR2E3 wild-type and mutant full-length proteins. COS-1 cells were transfected with various combinations of Gal4 (50 ng) and NR2E3 wild-type and mutant (100 ng) expression plasmids. Transcriptional activity of a NR2E3 responsive reporter gene was measured. Normalized values are expressed as relative luciferase activity.

In order to test the possibility that NR2E3 is behaving differently on inactivated and activated promoters, we tested the four mutants for their ability to repress transcription driven by Gal4 activation (Figure 5). The activation by Gal4 is resulting from a cryptic Gal4 binding Element beside the NR2E3 responsive element in the reporter construct used [14]. Activation (two-folds) was observed in the presence of Gal4 protein. This activation was repressed by wild-type NR2E3. The ESCS mutants have similar inhibitory properties toward this Gal4-mediated transcriptional activity.

## Discussion

The transcriptional inhibitory property of NR2E3 was also reported in other cell types, such as the human embryonic kidney (HEK) cells HEK 293, the kidney cells CV-1, and more important the retinal pigmented epithelium (RPE) cells RPE-J [14,22]. This inhibition was also observed when NR2E3 was tested as a full-length protein on a selected DNA binding element [14]. We have observed that the inhibition mediated by NR2E3 resembles that of unliganded RARα. This suggests the following: (1) that the inhibitory function of NR2E3 results from interactions of the LBD with co-repressors; (2) that only a conformational change, may be induced by binding to a ligand not present in these cells; (3) the exchange of co-repressors to co-activators could results in transcriptional activation [27]. Candidate ligands, as the all-trans and 9-cis retinoic acid and the 11-cis retinaldehyde, have been previously excluded [22]. Nevertheless, the 13-cis retinoic acid was recently reported as an NR2E3 agonist using a transcriptional activation assay [28].

**Figure 6:**
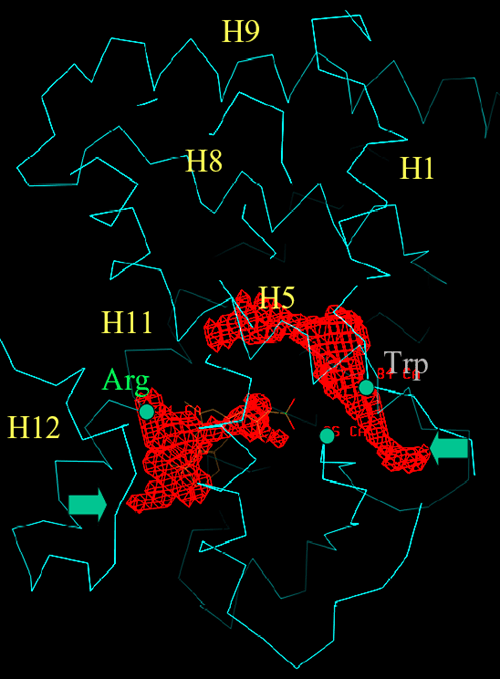
Molecular model of NR2E3^LBD^. Homology modeling of the NR2E3^LBD^ based on the RAR^LBD^ crystal structure. Highlighted are several residues that mutated in enhanced S-cone syndrome. The residue R385, shown in green, was predicted to localize in the ligand hydrophobic pocket. Mutation of W234, shown in red, was predicted to modify the ligand pocket conformation.

We have demonstrated here that some of the NR2E3 mutants that cause ESCS disease are not defective in transcriptional inhibitory activity. Four mutant proteins (E121K, W234S, R309G, and R311Q) retain transcriptional repression when tested as Gal4-fusion. Two of these mutants (W234S and R311Q) that tested as full length on the identified NR2E3 responsive element are also fully capable of repressive function. The activation by Gal4 is resulting from a cryptic Gal4 binding Element beside the NR2E3 responsive element in the reporter construct used (M407K and R385P). This absence of correlation was also observed for Gal4 activated transcriptional activation (Figure 5). A molecular model of NR2E3^LBD^ was established from the RAR^LBD^ crystal structure (Figure 6) [33]. The importance of position of M407K in the α-helix H12 of nuclear receptors that interacts with co-regulators [30] is suggested by the loss of repressive activity of the artificial mutant with a deletion of that helix (DH12). The position of the R385P mutation within a predicted hydrophobic pocket in a structural model of the LBD of NR2E3 might suggest the requirement of that residue in the interaction with a putative activating ligand [28]. It is unclear why the artificial mutant R385L retains transcriptional repression in COS-1 but not in HeLa cells.

The work presented here demonstrates that there is no correlation between the transcriptional inhibition mediated, in vitro, by the ligand binding domain of NR2E3 and the phenotype of ESCS. The difference between the ESCS mutants observed is not the result of differential interactions with protein partners such as the nuclear receptor NR1D1 or the homeoprotein Crx reported to involve the DBD of NR2E3 [15,23].

There is an ongoing debate about the mechanisms leading to excess of S-cones in ESCS. The models currently discussed involved the inhibition of S-cone specific genes by NR2E3 with [15,23,24] or without activation of rod-specific genes [14]. The absence of rod function in ESCS [3,4] argues for the involvement of NR2E3 in regulating rod-specific genes, while the absence of perturbation of rod-specific expression in the *rd7* retina [14] indicates that in the absence of NR2E3, rod-specific genes are expressed at a normal level.

While our results do not address the mechanisms behind the lack of correlation between NR2E3 mutations and ESCS the results point to the possible existence of transcriptional activation properties of NR2E3 regulated by a yet to be identified ligand. ESCS mutants might all be defective in transcriptional activation in addition for some of them to reduced transcriptional inhibition. The recent identification of NR2E3 agonists is an element supporting this hypothesis.
